# A FACS-based approach to obtain viable eosinophils from human adipose tissue

**DOI:** 10.1038/s41598-020-70093-z

**Published:** 2020-08-06

**Authors:** James D. Hernandez, Ben Yi Tew, Ting Li, Gerald C. Gooden, Hamza Ghannam, Mia Masuda, James Madura, Bodour Salhia, Elizabeth A. Jacobsen, Eleanna De Filippis

**Affiliations:** 1 Division of Endocrinology, Diabetes and Metabolism, College of Medicine, Mayo Clinic Arizona, 13400 East Shea Boulevard, Scottsdale, AZ 85259 USA; 2 Department of Translational Genomics, Keck School of Medicine, University of Southern California, Los Angeles, CA USA; 3 Division of General Surgery, Mayo Clinic Arizona, Scottsdale, AZ USA; 4 Division of Allergy, Asthma and Clinical Immunology, Mayo Clinic Arizona, Scottsdale, AZ USA

**Keywords:** Biological techniques, Cell biology, Immunology, Physiology, Endocrinology, Medical research

## Abstract

Eosinophils have been widely investigated in asthma and allergic diseases. More recently, new insights into the biology of these cells has illustrated eosinophils contribute to homeostatic functions in health such as regulation of adipose tissue glucose metabolism. Human translational studies are limited by the difficulty of obtaining cells taken directly from their tissue environment, relying instead on eosinophils isolated from peripheral blood. Isolation techniques for tissue-derived eosinophils can result in unwanted cell or ribonuclease activation, leading to poor cell viability or RNA quality, which may impair analysis of effector activities of these cells. Here we demonstrate a technique to obtain eosinophils from human adipose tissue samples for the purpose of downstream molecular analysis. From as little as 2 g of intact human adipose tissue, greater than 10^4^ eosinophils were purified by fluorescence-activated cell sorting (FACS) protocol resulting in ≥ 99% purity and ≥ 95% viable eosinophils. We demonstrated that the isolated eosinophils could undergo epigenetic analysis to determine differences in DNA methylation in various settings. Here we focused on comparing eosinophils isolated from human peripheral blood vs human adipose tissue. Our results open the door to future mechanistic investigations to better understand the role of tissue resident eosinophils in different context.

## Introduction

The emergence of a new branch of biological science, deemed immunometabolism^1^, which explores the interaction between tissue resident immune cell populations and the metabolism of the tissue itself, has expanded the understanding of eosinophil biology much beyond the age-old dogma of host defense against parasitic infestation and as inflammatory mediators in asthma^2,3^. It is now known that eosinophils have multiple functions in many tissues including roles in remodeling during development, defense against pathogens, and regulation of other immune cells (reviewed in^4–6^). Recently, eosinophils have been shown to regulate glucose homeostasis in adipose tissue by maintaining an anti-inflammatory state within healthy fat of rodents^7^. As the field of immunometabolism grows and begins to translate to human studies, it is essential to have access to pure isolated populations of the individual cell types being studied. Although methods for immune cell isolation and characterization are available and described for adipose tissue in mice^8,9^, methods for isolation from human fat are limited, particularly for eosinophils. This has proven to be a difficult proposition when considering (1) the relative scarcity of all immune cell types found within human adipose tissue, (2) the difficulty in obtaining human samples, (3) the lack of current available protocols describing the isolation of eosinophils from human adipose tissue, despite protocols that exist for the study of various cell types in human fat^10,11^.

To date, isolation of viable human eosinophils from blood can be completed by density gradient or by antibody mediated depletion (e.g., magnetic depletion) of other leukocytes to avoid excessive manipulation of eosinophils^12–14^. These methods, while favorable for blood-derived eosinophil isolation, remain problematic for tissue-derived eosinophils, which require enzymatic degradation of tissue and inclusion of more diverse cell compositions within the digested sample. Flow cytometric analysis has provided insight into the presence of eosinophils and cell surface activation state of tissue eosinophils^15,16^. However, a mere description of their presence or number in the adipose tissue although important, is not comprehensive. As of today, techniques to isolate adipose tissue-resident eosinophils for further ex vivo analysis are not well described in the literature, significantly limiting our understating of the functional role that these cells have in health and disease states like obesity and insulin resistance. A more complete understanding of their function and/or their impairment could potentially harbor important and innovative targets for drug therapy development.

Therefore, our focus was to employ fluorescence-activated cell sorting (FACS), which is a useful, rapid method for isolating pure cell populations from a mixed population based on size, granularity and fluorescence of labeled antibodies to specific cell surface proteins^17^. This method has been used to sort eosinophils from blood^18^ and bronchoalveolar lavage^19^, but to our knowledge, not from any other organ tissue for further downstream analysis. As such, we sought to develop a method to isolate eosinophils from small amounts of biopsied human adipose tissue with the goal of performing downstream analysis of nucleic acids from isolated cells.

Differential gene expression is often measured through transcript analysis via microarray or RNAseq. High quality RNA isolation from eosinophils derived from peripheral blood has been described^20^ and indeed we have succeeded in this procedure as well, however, this was accomplished with, at minimum, a full order of magnitude greater cell number than recovered from our adipose tissue samples. Given the multitude of inherent ribonucleases found within eosinophils^21^ coupled with the scarcity of the cells in tissue, these techniques prove problematic for human tissue-derived eosinophils and RNA transcript analysis is not pursued^20^. To circumvent this limitation, we choose to address our focus toward an epigenetic evaluation of eosinophils. Epigenetic regulation is exerted through a plethora of chemical modifications including histones modifications and DNA methylation and plays a prominent role in a number of complex diseases^22^. Epigenetic analysis of human circulating eosinophils^23,24^ has been reported and comparisons of DNA methylation patterns in asthmatic^25^ and diabetic^26^ patients has been studied, suggesting this avenue would be insightful for adipose tissue-derived eosinophils. In the current study, we describe a process to successfully analyze the genome of eosinophils derived from human adipose tissue via assessment of DNA methylation using whole genome bisulfite sequencing^27^ (WGBS). To our knowledge, this represents the first opportunity to evaluate gene regulation in these cells while overcoming the known limitation in analyzing the transcriptome of human adipose tissue eosinophils. Overall regulation of gene expression secondary to epigenetic marks has been defined in many fields of biology and medicine, including inflammation^28^ and obesity^29^.

## Results

### Immunostaining detects eosinophils resident in different types of human adipose tissue

**Figure 1:**
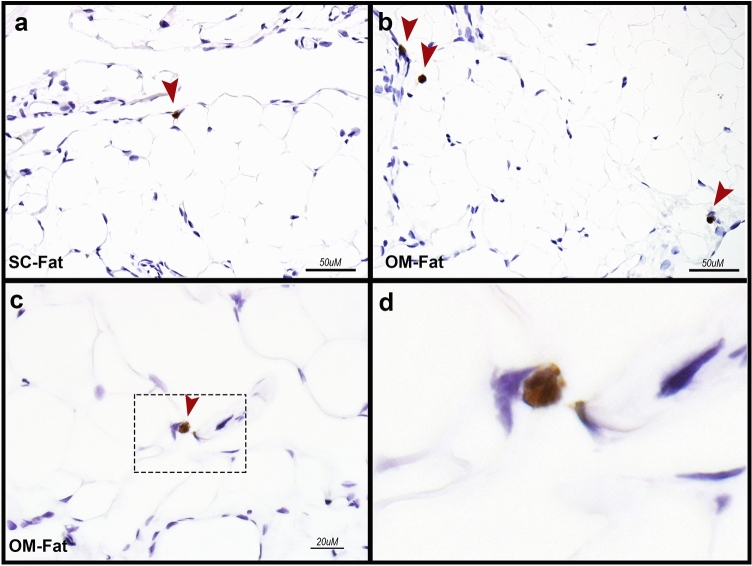
Immunohistochemistry of eosinophils in human adipose tissue. Immunohistochemical staining (brown cells) with antibody to eosinophil peroxidase specifically stains eosinophils within adipose tissue. (**a**) Subcutaneous fat, scale bar = 50 µM (SC-Fat), (**b**) Visceral fat (OM-Fat), scale bar = 50 µM, (**c**) Visceral fat (OM-Fat), scale bar = 20 µM. Dotted rectangular and arrow point to eosinophil, (**d**) Visceral fat (OM-Fat), enlarged rectangular area depicted in (**c**) on further enlargement identifies eosinophil.

To demonstrate the presence of adipose tissue-resident eosinophils in situ, we completed immunostaining assessment of different kinds of human adipose tissue. Eosinophils are difficult to identify by standard hematoxylin–eosin staining, particularly in adipose tissue. Therefore, we used an antibody to eosinophil peroxidase (EPX) that is unique to eosinophils to identify these cells in two types of adipose tissue, subcutaneous (SC) fat and omental (OM) fat. As shown in Fig. 1, eosinophils localized to the adipocyte regions of fat. As these cells are scarce for counting by immunohistochemistry and our goal was to isolate eosinophils for further downstream analysis, we performed FACS to isolate pure populations of eosinophils.

### Gating strategies including Siglec-8 and CD66b identified eosinophils in blood and human adipose tissue

**Figure 2:**
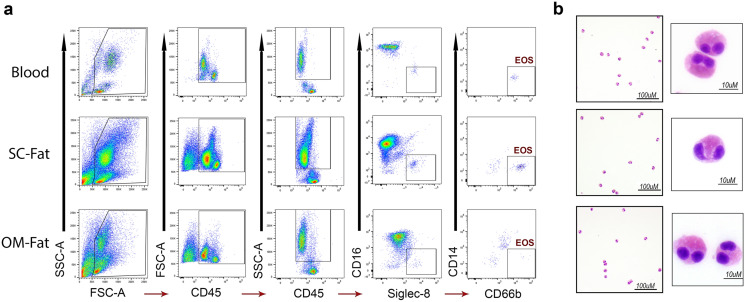
FACS gating scheme for eosinophil sorted from human blood, SC-Fat, and OM-Fat. (**A**) Samples underwent gating to remove doublets and dead cells (not shown), followed by gating on forward (FSC) and side scatter (SSC). Leukocytes were identified as CD45 (ubiquitous leukocyte marker), SSC hi to gate for granulocytes. Next, CD16-Siglec-8^+^ cells were selected, followed by CD14-Siglec8^+^ cells to provide maximal separation of populations with these fluorochrome conjugated antibodies. The eosinophils (EOS) population was identified as CD45^+^CD14^−^CD16^−^CD66b^+^Siglec-8^+^and were sorted to ≥ 99% purity. (**B**) Representative Hema 3 stained cytospins of sorted populations show eosinophils at 20× and 60× magnification.

As eosinophils have not been characterized by flow cytometry in human fat, we first determined the repertoire of antibodies required to successfully sort eosinophils. Our gating strategy relied on antibodies used previously for identification of eosinophils in blood through exclusion of CD14 and CD16 and inclusion of Siglec-8 and CD66b^15,30,31^ (Fig. 2a). Eosinophils were identified as CD45^+^CD14^−^CD16^−^CD117^−^Siglec-8^+^CD66b^+^. The profile of expression of Siglec-8 appears to be similar between blood and fat, while CD66b has a higher mean fluorescent intensity in adipose tissue as compared to blood, potentially indicating a different basal state of activation for eosinophils in fat^31^. From both SC and OM fat samples we first separated the stromal vascular fraction (SVF) composed initially of a mixed cell population, from which we were able to isolate pure eosinophils (Fig. 2b) after applying our gating strategy.

### Siglec-8 is able to identifies mast cells from human adipose tissue

**Figure 3:**
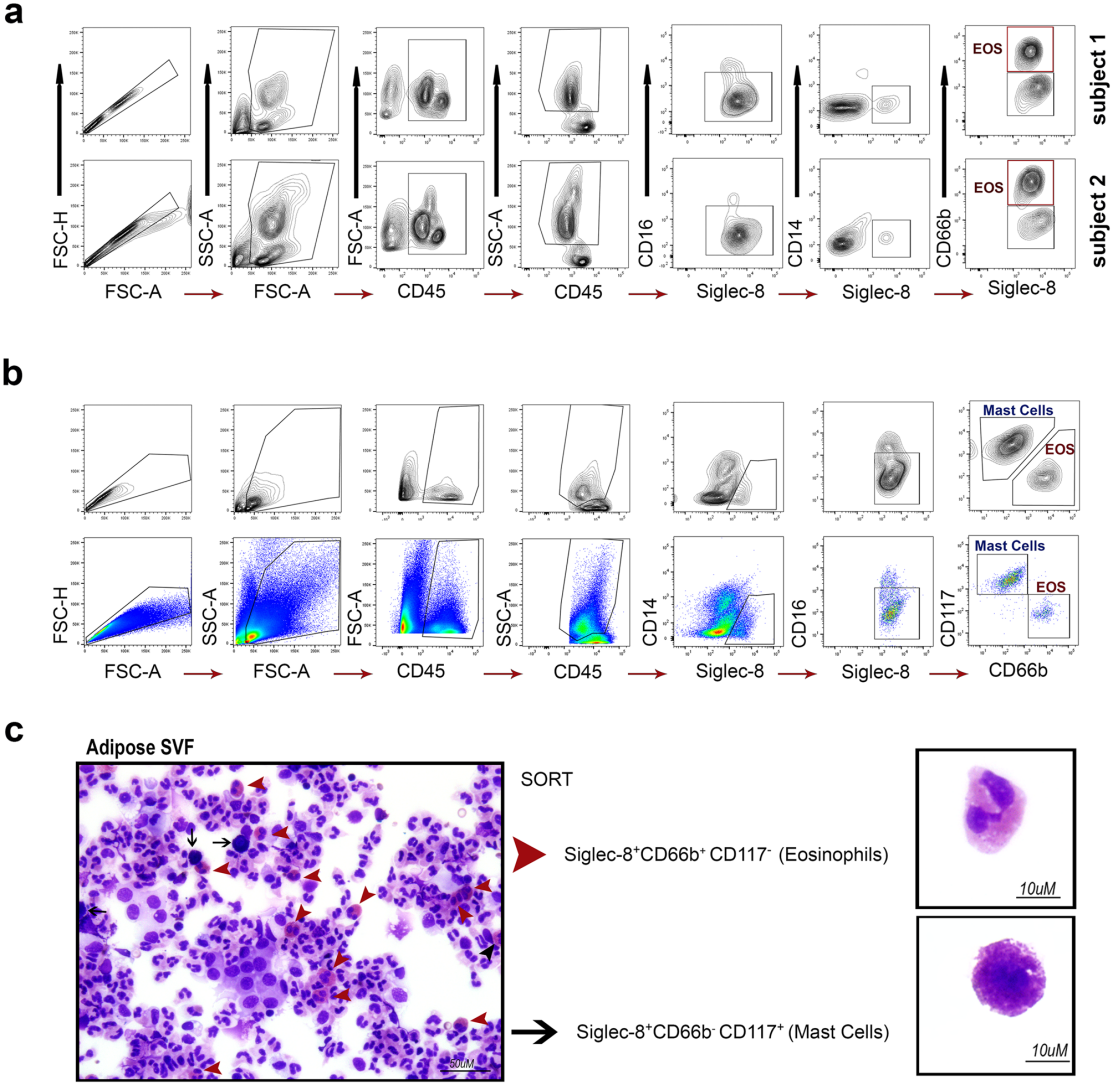
Eosinophils can be sorted similarly from adipose tissue and differentiated from mast cells in the same tissue. (**a**) Similar to the gating strategy in Fig. 2, eosinophils (EOS) were identified as CD45^+^CD14^−^CD16^−^CD66b^+^Siglec-8^+^ from two subjects. (**b**) To identify the adipose Siglec-8^+^CD66b^−^ negative population seen in (**a**), we added CD117 to the cocktail and adjusted the gating strategy. Mast cells are CD45^+^CD14^−^CD16^−^CD66b^−^Siglec-8^+^CD117^+^and Eosinophils are CD45^+^CD14^−^CD16^−^CD66b^+^Siglec-8^+^CD117^−^ in this gating strategy. (**c**) As shown by Hema 3 staining of pre-sort SVF and post-sort SVF cytopspins, both eosinophils and mast cells are present in the SVF fraction they can be sorted to 99% purity with this gating strategy.

Interestingly, from human adipose tissue depots we noticed a population of cells not present in blood that was Siglec-8^+^ and CD66b^−^ (Fig. 3a). As Siglec-8 binds eosinophils^32^, basophils^33^ and mast cells^32^, we tested the CD66b^−^ population, which excludes basophils, and the surface marker C-kit (CD117) for mast cells (Fig. 3b). The gating strategy and purified sorted populations confirmed eosinophils are Siglec-8^+^CD66b^+^CD117^−^ and mast cells are Siglec-8^+^CD66b^−^CD117^+^, representing a novel method of sorting both mast cells and eosinophils from human adipose tissue. As shown in Fig. 3c, The SVF contains both mast cells and eosinophils, and we were able to sort these two populations out separately with ≥ 99% purity.

### EMR1 as a potential alternative to Siglec-8 method for isolation of human adipose tissue eosinophils

**Figure 4:**
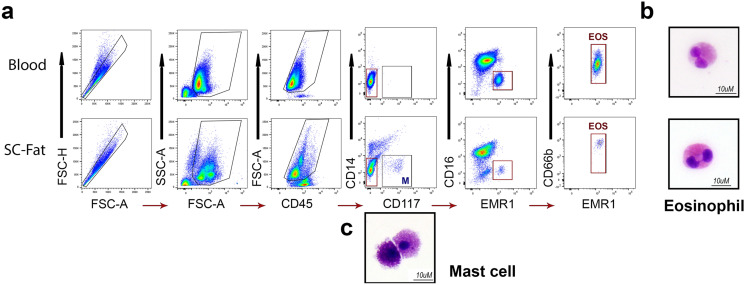
Eosinophils isolated with EMR1 gating strategy. (**a**) To isolate eosinophils by an alternative labelling and gating strategy we replaced Siglec-8 with EMR1. Blood and adipose tissue were completed side by side for comparison. Eosinophils were CD45^+^CD14^−^CD16^−^CD117^−^EMR1^+^CD66b^+^ and mast cells were readily defined by CD45^+^CD14^−^CD117^+^. (**b**) Representative images of > 99% pure sorted eosinophils and mast cells are shown by Hema 3 staining.

Although antibodies to Siglec-8 are very useful for identifying eosinophils, binding of antibodies to Siglec-8 has been shown to induce eosinophil cell death in human cells that are pre-treated with type 2 cytokines such as IL-5 or IL-33^34^ even without secondary antibody ligation^35^. The antibody (79C) matches the same epitope as the reported antibodies (2C4 and 2E20)^36^ known to mediate this cell death. Furthermore, the milieu and the pre-conditioned state of adipose tissue derived eosinophils remains unknown to us. These combined factors could potentially initiate intrinsic cell death. This is not problematic for immediate downstream molecular analysis such as epigenetics, however, it could greatly affect the ability to complete any additional ex vivo assays. Thus, we wished to demonstrate an additional means to isolate adipose tissue-derived eosinophils. As an alternative to Siglec-8, we tested an antibody to EMR1, shown to be specific to eosinophils^37,38^ which does not induce cell death with direct ligation^38^. Adopting this gating strategy, we were able to distinguish a pure eosinophil population from mast cells, such that eosinophils were CD45^+^CD14^−^CD16^−^CD117^−^EMR1^+^CD66b^+^ (Fig. 4a–c). In the adipose tissue, the gating strategy indicates that all EMR1^+^CD66b^+^ are likely to be the same eosinophils as the Siglec8^+^CD66b^+^ population as EMR1 is reported to recognize all Siglec8 + eosinophils^37^.

With all these methods we were able to obtain from 2 g of human adipose tissue up to 10^4^ EOS at ≥ 99% purity as determined by post sort FACS and Hema 3 staining of cytospins. This is in comparison to the isolation of 2–5 × 10^5^ eosinophils from 20 ml of whole blood, demonstrating the differential in numbers of eosinophils in fat as compared with blood.

### Downstream WGBS analysis of isolated adipose tissue-derived and blood eosinophils

The isolated eosinophils were subjected to DNA extraction. The extracted DNA was of high quality as determined by TapeStation, with an average DNA integrity (DIN) score of 8.02, out of a maximum of 10, suggesting that the DNA was of high molecular weight and not degraded. In order to assess the viability of analyzing the DNA from isolated eosinophils, we performed WGBS, which measures the DNA methylation levels of CpG loci across the entire genome. Comparative eosinophil gene expression studies utilizing methylation patterns have been described in cells isolated from peripheral blood^23,24^, however, studies on eosinophils isolated from any other tissue are lacking. From the WGBS results, there was an average alignment efficiency of 78.2% across all samples using the Bismark aligner^39^, which is optimized for bisulfite sequencing. Furthermore, 89.7% of the human genome was covered by the sequencing. This again reflects the high integrity of the extracted DNA and their suitability for next-generation sequencing analysis.

**Figure 5:**
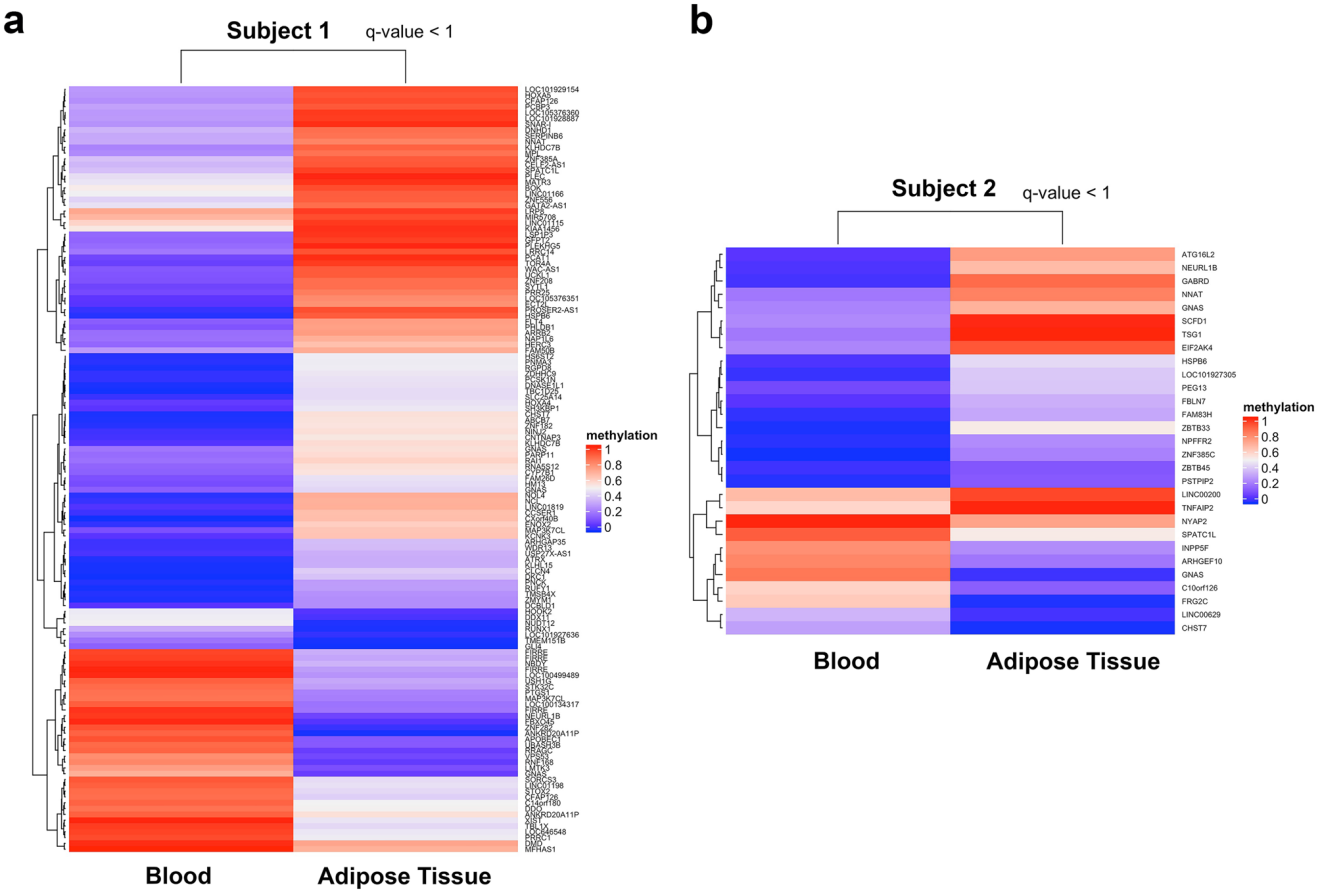
Heatmaps of statistically significant differentially methylated regions (DMRs) identified from eosinophils isolated from different sources using WGBS data. **a**,**b** DMRs from eosinophils isolated from peripheral blood (Blood) versus adipose tissue (AT) in subject 1 (**a**) or subject 2 (**b**).

Using the WGBS results, differentially methylated regions (DMRs) were identified between different sets of eosinophils (Fig. 5a,b). Here we report the DMRs found between eosinophils extracted from the adipose tissue and peripheral blood of two subjects (Fig. 5a,b). A total of 132 DMRs were found between blood vs. adipose tissue derived eosinophils in subject 1, most of them being hypermethylated (90 hypermethylated and 42 hypomethylated). In contrast, 29 DMRs were found between the eosinophils extracted from the adipose tissue and peripheral blood of subject 2, most of them being hypomethylated (9 hypermethylated and 20 hypomethylated). Of these DMRs, an average of 78.3% were found on or adjacent to CpG islands, which are regions with high CpG density and are frequently found in regulatory regions of genes. One gene, in particular, Neuronatin (NNAT), was hypermethylated in adipose tissue as compared to blood eosinophils for both subjects. Interestingly, this gene is associated with metabolic changes in humans^40,41^ and is found expressed in eosinophils in human RNA^42^ arrays.

These results demonstrate that the eosinophils are not disrupted during the isolation process, and suggest that biologically relevant differences in methylation status can be identified from eosinophils derived from different sources.

## Discussion

The goal of this study was to develop a method to consistently obtain viable eosinophils from whole human adipose tissue suitable for downstream molecular analysis. The need for such analysis arises from recent data suggesting that eosinophils help preserve insulin sensitivity^7^ in animal models of diet-induced-obesity. A limitation in the potential translation of the immunometabolic function(s) of eosinophils is due to lack of a robust method to isolate viable eosinophils from human adipose tissue for downstream analysis. Utilizing cell surface markers that differentiate eosinophils from mast cells, and other tissue resident immune cells, we were able to isolate pure eosinophils from adipose tissue for downstream analysis. Cell surface markers Siglec-8 and CD66b were sufficient for separation of eosinophils from mast cells as confirmed by staining with CD117. To compliment the Siglec-8 targeting method we devised a strategy utilizing EMR1 in combination with CD117 and CD66b.

DNA extracted from these cells allowed DNA methylation analysis through WGBS, comparing DNA methylation levels of CpG loci across the entire genome from eosinophils derived from various tissues. While DNA methylation is not static, it is also not an immediately dynamic process^43^. We have completed the isolation of blood and tissue derived eosinophils within a standard time frame to avoid introducing artifact in the methylation asset of our samples. Moreover, long-term storage of samples does not appear to significantly modulate the DNA methylation of samples, even under different conditions, indicating it is not as labile as RNA or protein^44^. The eosinophils recovered from both tissues were then successfully profiled using WGBS. The high yield and integrity of the DNA indicates that other genomic techniques (i.e. Chip-seq or ATAC-seq)^22^ could also be used to profile eosinophil phenotypes. This approach aligns with recent studies demonstrating FACS sorted blood purified eosinophils from asthmatics provides sufficient quality for identifying key methylation changes between asthmatics and normal patients^23^. Indeed this study highlighted the need for rare and difficult to obtain cells kinds like eosinophils that epigenetic studies should be the future avenue to define their various functional state^45^.

To date we are unaware of any epigenetic studies on eosinophils isolated from human tissues, and in particular on human adipose tissue-derived eosinophils. Although there are examples of epigenetic studies completed on adipose tissue, they lack information on tissue resident eosinophils possibly due to their low number^46^. Thus, FACS sorting of tissue eosinophils is likely a critical step needed for future studies.

Whole genome or whole exome sequencing would be ideal for studying eosinophil-derived diseases such as eosinophilic leukemia or eosinophilic esophagitis, where mutations have been identified as potential contributors to disease progression^47^. With single cell sequencing technology becoming more advanced, our FACS-based method of isolating eosinophils can be directly fed into plate-based single cell DNA, or into other microfluidics-based protocols. Based on the DMR found between blood and adipose tissue, it is possible to speculate for this approach to be useful for assessing molecular alterations and associated signaling pathways changes of tissue resident eosinophils. For example, we identified, albeit with only two subjects, that metabolic associated protein NNAT was differentially methylated in fat as compared to blood.

Our study has a few identified limitations. To begin, the limited amount of adipose tissue obtained (2–10 g) effectively capped the overall number of eosinophils we were able to isolate. Because of the limited number of cells collected we could not perform additional functional studies such as RNAseq or culture for chemotaxis assays. For those studies, as currently demonstrated^18,48^, anywhere from 5 × 10^4^ cells per condition up to 1 × 10^6^ cells total are required. Our recovery of up to 10^4^ eosinophils on small amount of tissue (2 g) fell orders of magnitude below the numbers required to conduct such experiments. Additional studies with larger starting material (i.e. adipose tissue samples collected during abdominoplasty or panniculectomy) could become an ideal model to isolate the required number of cells to perform functional assays. However, these assays were beyond the scope of the current project, which was to develop a reliable method to isolate viable eosinophils from human adipose tissue and determine an adequate utility for comparative downstream analysis.

Overall, our results open another avenue to explore the immunometabolic function of adipose tissue resident eosinophils. We clearly demonstrated the ability of our technique to isolate and distinguish eosinophils (and if desired, mast cells) from other immune cells derived from human adipose tissue, opening the door for further metabolic characterization of these cells in varying microenvironments. Future studies using our method and larger sample size are required to in order to draw conclusions on definitive DNA methylation changes. This, in turn, could lead to identification of novel targets for development of innovative therapeutic interventions.

## Methods

### Adipose tissue collection

Mayo Clinic Institutional Review Board (IRB) approval was obtained for this study and the informed consent form. In accordance to Mayo Clinic research policies, our study was registered at https://www.clinicaltrial.gov (NCT02378077). Before entering the study, all subjects provided written informed consent to participate, in accordance with the Declaration of Helsinki. In addition, all study related procedures were performed in compliance with Mayo Clinic’s policies, rules, ethical standards and regulation for research conduct. As part of the study design, the collection of adipose tissue (AT) from healthy subjects occurred in patients undergoing elective abdominal surgery at Mayo Clinic Arizona. Through collaboration with the general and gynecology surgical departments, we collected a wide range of human SC and OM fat (2–10 g). All subjects recruited in this study were screened for atopic syndrome, asthma, allergies, and tobacco use. No glucocorticoid, antihistamines or other allergy medications were allowed in this study. We specifically chose to enroll a group of volunteers without any abnormalities in the peripheral blood counts, confirmed by normal complete blood counts. A urine pregnancy test in pre-menopausal subjects was also completed. Upon collection of the fat, tissue was placed in 25 ml DMEM low glucose with l-glutamine and pyruvate (Gibco 11885-084) and transported on ice to the laboratory for processing.

### AT-Immunohistochemistry for eosinophils

A portion of SC-fat or OM-fat was formalin fixed and paraffin embedded. Five-micron slices underwent immunohistochemistry using monoclonal mouse anti-eosinophil peroxidase (EPX-mAb; clone 82.2.1^20,49^, a kind gift of Dr. E.A. Jacobsen, Mayo Clinic Arizona) with modifications. Slides underwent heat induced antigen retrieval with pH 6.0 citrate buffer (Vector H-3300) using variable (hi-power output for 2 min and low-power output for 14 min) microwave settings followed by a 30 min room temperature period to cool. After washing with distilled water 3 times, slides were quenched in 3% hydrogen peroxide for 10 min, followed by 3 washes with Dasko Wash Buffer (Dako S3006). Endogenous enzymes and proteins were blocked by applying Dako Dual Endogenous Blocking Solution (Dako S2003) for 10 min at room temperature followed by 5% normal goat serum diluted in Dako Wash Buffer for 1 h at room temperature. EPX antibody prepared in Antibody Diluent with Background Reducing Components (Dako S3022) was applied at a 1:1,000 dilution (1 µg/ml final concentration) and incubated for 30 min at room temperature. Slides were washed with Dako Wash Buffer followed by the addition of secondary antibody (ImmPRESS HRP Anti-mouse IgG (Peroxidase) Polymer made in Goat (Vector MP-7452)) for 30 min at room temperature. Slides were washed three times with Dako Wash Buffer and developed with DAB Peroxidase (HRP) Substrate Kit (Vector SK 4100) for 10 min at room temperature. After washing with distilled water, slides were counterstained with hematoxylin (Dako S3302) cleared with glacial acetic acid rinses followed by distilled water rinses. Slides were dehydrated with increasing concentrations of ethanol and mounted with non-aqueous mounting media (ClearMount, ThermoFisher).

### AT-Stromal vascular fraction (SVF) preparation

Approximately 50% of the original biopsy sample (ranging from 2 to 5 g) was used to isolate SVF through a combination of manual disruption, liberase digestion, and mechanical sieving. Briefly, the sample was first diced with dissection scissors then minced with razor blades in a petri dish in a digestion solution of low glucose DMEM and 100 µg/ml Liberase (Roche), 3 ml per gram tissue. The minced tissue was placed in a 15 ml polypropylene tube and incubated at 37 °C for 45 min on a rotating wheel. The digested tissue was then passed through a 300 µm metal sieve and washed with an equal volume of Dulbecco's Phosphate buffered saline (DPBS) without calcium (Ca^2+^) and magnesium (Mg^2+^) (Sigma-Aldrich,). The sieved pass through was then placed in fresh 15 ml tubes and centrifuged at 400*g* for 5 min, 4 °C. After centrifugation, the top oil layer was aspirated, the floating adipocyte layer was collected and flash frozen for future evaluation, and the aqueous layer was then aspirated leaving the SVF pellet. The pellet was resuspended and washed in 5 ml PBS with 2w/v% BSA (Sigma) then centrifuged at 400*g* for 5 min. This was repeated twice. The pellet was then suspended in 1 ml cold water for 20 s for erythrocyte lysis and 1 ml of 2× PBS was added to return to an isotonic state. The cells were then passed through a 5 ml polystyrene cell-strainer cap tube (40 µm pore) and centrifuged for 5 min × 400*g*. The supernatant was discarded and the resulting pellet was suspended in 1 ml PBS and placed on ice.

### Isolation of eosinophils from AT-SVF by FACS

Immediately after suspending the SVF pellet in PBS, 1 µl eBioscience Fixable Viability Dye eFluor 455UV (Invitrogen Cat# 65-0868-14) per 1 × 10^6^ cells was added to the tube and incubated for 30 min at 4 °C in the dark. Cells were then washed with PBS, centrifuged and suspended in FACS staining buffer (Invitrogen Cat# 00-4222-26) at 1 × 10^6^ cells/ml. To this, 10 µl mouse anti-human CD14 FITC-conjugated antibody (clone M5E2 BD Pharmingen Cat# 555397), 10 µl mouse anti-human CD45 APC-conjugated antibody (clone HI30 BD Pharmingen Cat# 555485), 5 µl mouse anti-human CD16 eFlour450-conjugated antibody (clone eBioCB16 Invitrogen Cat# 48-0168-42), 5 µl mouse anti-human CD66b PE-Cyanine7-conjugated antibody (clone G10F5 Invitrogen Cat# 25-0666-42), 5 µl mouse anti-human Siglec-8 PE-conjugated antibody (clone 7C9 BioLegend Cat# 347104), or 10 µl hamster anti-human EMR1 RPE-conjugated antibody (clone A10 Bio-Rad Cat# MCA2674PE), or 5 µl mouse anti-human CD117 Brilliant Violet 650-conjugated antibody (clone 104D2 BioLegend Cat# 313222) were added to the tube per 1 × 10^6^ cells and incubated for 30 min at 4 °C in the dark with occasional gentle agitation (Table 1). Cells were then washed with 5 ml DPBS, centrifuged 5 min × 400*g* at 4 °C and the supernatant was discarded. The stained cell pellet was resuspended in 1 ml of cold FACS buffer and kept on ice until sorting.

**Table 1:** Antibody information for FACS staining of human adipose tissue.

Antigen	Vol (µl)/ml/1 million cells	Fluorochrome	Vendor	Clone	Catalog
Live/dead	NA	UV 455	Invitrogen	None	65-0868-14
CD45	10	APC	BD Pharmingen	H130	555485
CD14	10	FITC	BD Pharmingen	M5E2	555397
CD16	5	eFluor 450	Invitrogen	eBioCB16	48-0168-42
CD66b	5	PE-Cy7	Invitrogen	G10F5	25-0666-42
Siglec-8	5	PE	BioLegend	7C9	347104
EMR1	10	RPE	Bio-Rad	A10	MCA2674PE
CD117	5	Brilliant Violet 650	BioLegend	104D2	313222

Eosinophils were sorted with a FACSAria III (BD Biosciences) (nozzle size 100 μm, low flow rate, sample injection chamber set at 4 °C) through the various gating schemes as shown in Figs. 2, 3, 4 such that eosinophils are CD45^+^CD14^−^CD16^−^Siglec-8^+^CD66b^+^CD117^−^ or CD45^+^CD14^−^CD16^−^EMR1^+^CD66b^+^CD117^−^. These gating schemes resulted in a cell population > 99% eosinophils as determined by post sort FACS and cytospin (500 rpm for 5 min CytoSpin 4 Cytocentrifuge, Thermo Fisher Scientific). Slides were air dried and stained with Hema3 staining set (Fisher) to validate eosinophil morphology. Trypan blue staining determined ≥ 95% viability of adipose tissue eosinophils isolated through this protocol. Controls for positive/negative cut-off for gating included fluorescence minus one (FMO) for each antibody in the panel (not shown). The reproducibility and feasibility of our protocol was tested by repeating every step enlisted multiple times with 5 different patients and on different days. Pseudocolordot-plots or density contour plots were completed using FlowJo^50^ version 10.5.3 (Ashland, OR).

### Isolation of eosinophils from peripheral blood

In parallel with adipose tissue isolation, 20 ml of whole blood was collected for eosinophil isolation. Granulocytes were concentrated through the use of Ficoll-Paque PLUS (density 1.077 g/ml) as per the product protocol^14^, and reported previously^14^. RBCs were lysed in water as described above and the pellet was incubated with the same antibody cocktail as described above for SVF. Cytospin slides revealed a cell population > 99% eosinophils, while Trypan blue staining determined ≥ 99% viability of peripheral blood eosinophils isolated through this protocol.

### Eosinophil DNA isolation and whole genome bisulfite sequencing (WGBS)

DNA was extracted from the isolated eosinophils using Quick-DNA/RNA Microprep Plus Kit (#D7005, Zymo Research). Sorted eosinophils were centrifuged 5 min × 400*g* at 4 °C and the supernatant was discarded. The cells were then lysed in 1 ml DNA/RNA Shield (Zymo Research) and DNA was extracted as per the kit protocol. Quantity and purity of the isolated DNA was determined by NanoDrop and by the 4200 Tapestation using Genomic DNA ScreenTapes (Agilent). Extracted DNA was used for whole genome bisulfite sequencing analysis (WGBS) as previously described^51^. Directional bisulfite-converted libraries for paired-end sequencing were prepared using the Ovation Ultralow Methyl-Seq Library System (NuGen), using the manufacturer’s suggested protocol. Bisulfite conversion was performed using the EpiTect Fast DNA Bisulfite Kit (Qiagen). Post-library QC was performed on the 4200 Tapestation using D1000 ScreenTapes (Agilent). Paired-end sequencing was performed on the Illumina Novaseq 6000 platform using the S1 flowcell for a total read length of 2 × 150 bp.

Bisulfite-modified DNA reads were trimmed for adapters using Trim Galore. Reads were then aligned to the bowtie2-indexed reference genome GRCh37 (hg19) using Bismark tool version 0.12.7^39^. Sequencing duplicates were removed using samblaster^52^. Methylation calling was performed using the Bismark “Methylation Extractor” module. Differentially methylated regions were identified using metilene^53^. Regions deemed statistically significant by metilene after adjustment for false discovery (q-value < 1) were used for subsequent analyses performed on R^54^.
