# Publisher Correction: A FACS-based approach to obtain viable eosinophils from human adipose tissue

**DOI:** 10.1038/s41598-021-98825-9

**Published:** 2021-09-24

**Authors:** James D. Hernandez, Ben Yi Tew, Ting Li, Gerald C. Gooden, Hamza Ghannam, Mia Masuda, James Madura, Bodour Salhia, Elizabeth A. Jacobsen, Eleanna De Filippis

**Affiliations:** 1 Division of Endocrinology, Diabetes and Metabolism, College of Medicine, Mayo Clinic Arizona, 13400 East Shea Boulevard, Scottsdale, AZ 85259 USA; 2 Department of Translational Genomics, Keck School of Medicine, University of Southern California, Los Angeles, CA USA; 3 Division of General Surgery, Mayo Clinic Arizona, Scottsdale, AZ USA; 4 Division of Allergy, Asthma and Clinical Immunology, Mayo Clinic Arizona, Scottsdale, AZ USA

Correction to: *Scientific Reports* 10.1038/s41598-020-70093-z, published online 06 August 2020

The original version of this Article contained an error in the spelling of the author Gerald C. Gooden which was incorrectly given as Gerald C. Gooden II.

The original Article has been corrected.

